# Auditory Rate Perception Displays a Positive Serial Dependence

**DOI:** 10.1177/2041669520982311

**Published:** 2020-12-22

**Authors:** Aysha Motala, Huihui Zhang, David Alais

**Affiliations:** The Brain and Mind Institute, University of Western Ontario, London, Ontario, Canada; School of Optometry and Vision Sciences, Cardiff University, Cardiff, UK; School of Psychology, The University of Sydney, Sydney, Australia; School of Psychological and Cognitive Sciences, Peking University, Beijing, China; School of Psychology, The University of Sydney, Sydney, Australia

**Keywords:** adaptation/constancy, audition, perception, temporal processing, time perception

## Abstract

We investigated perceived timing in auditory rate perception using a reproduction task. The study aimed to test (a) whether central tendency occurs in rate perception, as shown for interval timing, and (b) whether rate is perceived independently on each trial or shows a serial dependence, as shown for other perceptual attributes. Participants were well able to indicate perceived rate as reproduced and presented rates were linearly related with a slope that approached unity, although tapping significantly overestimated presented rates. While the slopes approached unity, they were significantly less than 1, indicating a central tendency in which reproduced rates tended towards the mean of the presented range. We tested for serial dependency by seeing if current trial rate reproductions depended on the preceding rate. In two conditions, a positive dependence was observed. A third condition in which participants withheld responses on every second trial produced a negative dependency. These results suggest separate components of serial dependence linked to stimulus and response: Withholding responses reveals a negative perceptual effect, whereas making responses adds a stronger positive effect that is postperceptual and makes the combined effect positive. Together, these data show that auditory rate perception exhibits both central tendency and serial dependence effects.

Accurate perception of the timing of events in our environment is essential for survival. Perceived timing can be impressively precise and accurate (Cicchini et al., 2012; Matthews & Meck, 2014), and yet it is also considerably malleable. Perceived timing is affected by adaptation aftereffects (Maarseveen et al., 2019; Walker et al., 1981) and by the nature of the stimuli carrying the timing information (Wearden et al., 1998; 2007). The temporal statistics embodied in prior probability distributions can be reshaped quite quickly (Roach et al., 2017) and there are surprising short-term adaptations of relative timing between auditory and visual stimuli from trial to trial (Van der Burg et al., 2013, 2015).

The finding that perceived audio-visual timing fluctuates between one trial and the next shows that successive timing judgments are not independent, exhibiting instead a serial dependence. Serial dependence refers to the tendency for perception on a given trial to be systematically biased by the stimulus or response from the immediately preceding trial (Fischer & Whitney, 2014). The topic of serial dependence has been very active recently with studies showing a positive serial dependence for a variety of perceptual attributes. For example, perception of basic visual features such as orientation and visual motion both show a positive serial dependence (Alais et al., 2017; Fischer & Whitney, 2014). More complex attributes such as numerosity and scene perception show a positive dependency (Cicchini et al., 2014; Corbett et al., 2011; Manassi et al., 2017), as do face gender and attractiveness, body shape, and eye gaze (Alais et al., 2018; Alexi et al., 2018; Corbett et al., 2011; Kok et al., 2017; Taubert et al., 2016; Xia et al., 2016). Even perception of more abstract attributes such as aesthetic ratings of artworks (Kim et al., 2019) show a positive dependence on the recent past. In all these examples, perception of the current stimulus is biased towards the previous stimulus (e.g., a neutral face will appear more attractive following an attractive face). Although this is a distortion of current perception, positive serial dependences are similar to perceptual priming effects and are thought to be beneficial by helping achieve perceptual stability (Cicchini et al., 2018; Kiyonaga et al., 2017) through the combination of current sensory input with recent input.

Not all serial effects are positive or “assimilative.” For instance, one recent study (Taubert et al., 2016) demonstrated that face expression exhibited a repulsive rather than attractive dependency on the preceding face, an effect more consistent with traditional repulsive perceptual aftereffects occurring after prolonged exposure to an adaptor. Other studies using sequences of brief stimuli have also found repulsive effects for visual orientation (Alais et al., 2017) and for perceived direction of auditory frequency sweeps (Alais et al., 2015). The functional advantage of a negative serial dependence is that it enhances discrimination around the adapted stimulus, improving our ability to perceive small differences. In the realm of temporal perception, audiovisual temporal order shows a negative serial dependence (Van der Burg et al., 2013; Van der Burg & Goodbourn, 2015; Van der Burg et al., 2015). In those studies, presenting an auditory-first asynchrony made a simultaneous stimulus more likely to be perceived as vision-first. This is a repulsive effect, similar to what is observed with classical perceptual aftereffects. Fischer and Whitney (2014) found a positive serial dependence in visual orientation perception for shorter stimulus durations, but a negative aftereffect with longer stimulus durations. Using a clever dual-task paradigm, Fritsche et al. (2017) have shown a repulsive effect for perception, but a positive serial dependency for perceptual decision. Together, the previous mixed findings of serial effects being negative or positive may be due to the weighted combination of repulsive perceptual and positive decisional effects, where perception is optimized for detecting changes and decision processes to form stable representations of the environment by integrating over longer times (Fritsche et al., 2017).

In this study, we test whether rate perception of isochronous tone sequences exhibits a serial dependency. Auditory rate is of interest because rhythms are ubiquitous in natural environments and there is a current debate regarding the relationship between auditory intervals and rate (Hartcher-O'Brien et al., 2016; Heron et al., 2012; Ivry, 1996; Johnston et al., 2006; Keele et al., 1989; Ono & Kitazawa, 2011). The presence of serial dependency effects may be helpful in elucidating the differences between the two. It is not clear what sign a dependency for auditory rate would have. Auditory tone sweeps show a negative dependency, as does audiovisual relative timing, yet there is also work showing that reproduction of temporal intervals shows a central tendency bias (Jazayeri & Shadlen, 2010). A central tendency effect is a bias whereby perceptual judgments are drawn towards the mean of a set of presented stimuli and under certain circumstances this can produce a data pattern similar to a positive dependency (Petzschner et al., 2015). Here we test for the presence of serial dependence in auditory rate perception using a simple rate reproduction paradigm. We predict that there will be a central tendency effect in the reproduced auditory rates, as found in previous timing research, but that we will also find evidence for serial dependence. By separating the central tendency effect, we will reveal whether serial dependence for auditory rate reproduction is positive (i.e., assimilative) and thus distinct from traditional repulsive adaptation effects.

## Methods

### Participants

A total of 20 subjects participated in the experiment (14 females and 6 males). Participants were required to have normal hearing and corrected-to-normal visual abilities. Participants were not excluded on the grounds of musical expertise. All experiments were conducted at the School of Psychology at the University of Sydney and written informed consent was obtained from all participants prior to their participation.

### Apparatus and Stimuli

Experiments took place in a sound-proof testing booth with subjects seated at a table in front of a timing reproduction device and a video monitor (Dell OptiPlex 7440, 24 in. monitor, running at 60 Hz) viewed from approximately 57 cm and a pair of loudspeakers. The auditory stimuli were amplitude modulations of a 500 Hz carrier frequency played a clearly audible and moderate sound pressure level of ∼76 dB(A). The amplitude modulations were sinusoidal and had a frequency drawn randomly from a uniform distribution spanning the range spanning 3–7 Hz. The auditory signals had a modulation index of 0.90 were presented within a background of quiet pink noise to mask soft sounds that penetrated the sound chamber from outside. Tone sequences were presented for a duration of 1 s, followed by a short pause drawn randomly from the range of 0.75–1.0 s during which a prominent red fixation cross was present on the screen. Following the pause, the cross became green, cueing the participant to respond. The response task was to reproduce the presented rate by tapping a small baton. The response baton had an accelerometer attached to the tip to trace its movement and participants tapped it back and forth against the sides of an aperture mounted on a wooden base to indicate the rate they perceived. The accelerometer’s output was recorded and the turning points in the oscillating trace where the baton bounced off the aperture’s edges were computed. The mean interval between reversals was calculated and the reproduced rate was recorded as the inverse of the mean interval duration. The standard deviation of the intervals was also recorded. The response window lasted 2 s and then after another short pause (random in the range of 0.25–0.35 s) the next trial began.

### Task and Procedure

The general experimental set-up presented subjects with a sequence of auditory rates, followed by a short pause and then a response period requiring the subject to reproduce the auditory frequency they perceived using a tapping response. The exact experimental details for each condition are described here.

#### Condition 1

As described earlier, a series of auditory rates was presented for 1 s that were drawn randomly from a continuous frequency range of 3–7 Hz and after a short pause of 0.75–1.0 s participants reproduced the rate they perceived using a response baton.

#### Condition 2

Condition 2 differed from Condition 1 in one respect: Participants only responded on every alternating trial. Although an auditory rate stimulus was presented on every trial, participants only responded on odd trials (1, 3, 5, etc.), as cued by a green fixation cross on the video monitor, and were not required to respond on the intervening even trials (2, 4, 6, etc.). During even trials, a red fixation cross was presented on the screen to remind participants not to respond.

#### Condition 3

Condition 3 was identical to Condition 1 except that the pace of the experiment was slower. This was to control for the slower response rate in Condition 2 where participants only responded to every second trial. In Condition 3, participants responded on every trial but the trials were presented at a rate corresponding to every second trial of Conditions 1 and 2. The slower rate was achieved by extending the pause before the participant’s response to 3.875 s. The red fixation cross was present throughout the extended pause.

Each subject completed three conditions and each involved two blocks of 80 trials, giving a total of 160 trials per condition. The order of experimental blocks was counterbalanced for each participant. The standard deviation of the intervals between each tap in the tapping response was used as an exclusion criterion to identify individual trials that had poor responses. Tapping that was regular had intervals with a low standard deviation and indicated a good response. At the trial level, excluding responses with a standard deviation of more than 150 ms was effective at capturing trials where the subject’s tapping response was either very erratic or simply skipped a beat. At the subject level, two subjects were removed entirely from the analysis because more than 50% of their trials were excluded for a certain condition. For the remaining subjects (*n* = 18), the group averaged percentages of excluded trials for the three conditions were 9.6%, 7.3%, and 9.1%, respectively.

## Experiment

The experiment comprised three conditions. The first involved a series of brief trials in which the participant heard an amplitude modulated auditory signal with a rate drawn randomly over trials from a range of 3–7 Hz. After each trial, the participant indicated the perceived rate using a reproduction task, as commonly used in studies of serial dependence and rate reproduction (Motala et al., 2018). If there is a positive serial dependence for auditory rate perception, as observed for a range of other perceptual attributes (Cicchini et al., 2014; Corbett et al., 2011), the data should show a positive relationship with the preceding trial’s rate, with reproduced rates being biased towards the rate presented on the preceding trial. In Condition 2, the stimulus presentation was identical but the participant only responded on every second trial. If a positive dependency is observed in Condition 1, Condition 2 will determine whether it is dependent on the response to the stimulus or to the stimulus itself. Withholding the response should remove a response-based serial dependency, eliminating the dependency or even reversing its sign to a negative dependency, as observed elsewhere (Van der Burg & Goodbourn, 2015). In Condition 3, the procedure is as for Condition 1 except that the interval between trials is extended so that they have the same timing as the “response” trials in Condition 2. Condition 3 should reveal the same pattern as Condition 1, as all trials are “response” trials, and any positive dependence should be enhanced by the longer interval between stimulus and response. This follows from a number of studies which have shown that positive dependencies tend to strengthen as the gap between stimulus and response increases (Bliss et al., 2017; Fritsche et al., 2017; Kanai & Verstraten, 2005). This could be explained by subjects having to hold the rates in working memory for a longer time (Bliss et al., 2017) and by a fading of repulsive adaptation from the stimulus as time elapses.

## Results

**Figure 1.**
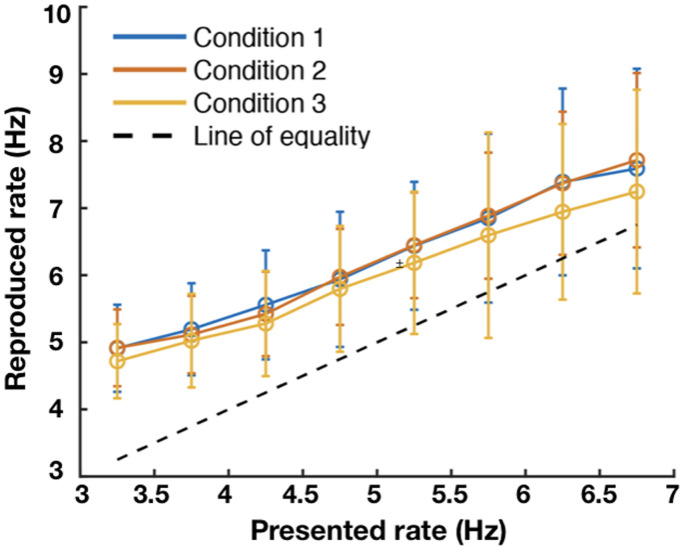
Group mean data from 17 participants showing reproduced temporal rate plotted against presented auditory rate, with error bars showing ±1 standard deviation. Presented rates were sampled randomly from a continuous range between 3 7 Hz and have been averaged into bins with a width of 0.5 Hz. The data from the three different conditions are remarkably consistent. First, all conditions show a significant elevation above the equality line (indicated by the dashed line), and the gain in reproduced rates is less than 1 (see Table 1).

For each participant, a linear regression model was fitted to examine the relationship between reproduced and presented rates in the three conditions. One participant was removed from the analysis because of very poor performance in one of the conditions (the function was virtually flat, with a coefficient less than 0.1), leaving 17 participants for the further analysis. The rate reproduction data for all three conditions are summarized in Figure 1 which shows group mean data (*n* = 17) as a function of the presented auditory rate. The presented rates varied continuously between 3 and 7 Hz and were binned into intervals of 0.5 Hz. If the reproduced rates matched the presented rates, the data points would lie along the equality line shown by the dashed diagonal line. It is clear that in general participants overestimated the auditory rate, and there was a tendency for the overestimation to diminish as the presented frequency increased.

Next, we quantitatively examined the relationship between reproduced and presented rates in the three conditions with the linear regression model mentioned before. Each participant’s regression coefficients were obtained. The group mean slopes and *y*-intercepts are shown in Table 1 and were tested for significance using a two-sided Wilcoxon signed-rank test. The first notable point is that the intercepts are all significantly greater than zero. This means that in reproducing the presented auditory rate, participants significantly overestimated the presented frequency. As can be seen by eye from the results plotted in Figure 1, there is a systematic upwards shift of reproduced auditory rates. This pattern of overestimation is consistent with other studies of auditory temporal reproduction (Ganzenmüller et al., 2012; Motala et al., 2018, 2020). A second point, less evident from Figure 1 but still significant, is that the slopes were reliably less than 1. The average slope of 0.81 indicates that, in general, participants were well able to discriminate the presented auditory rates in their reproductions. However, a slope less than 1 means that the range of reproduced rates was compressed relative to the presented range, a feature indicative of central tendency which has been observed in others studies of temporal perception (Jazayeri & Shadlen, 2010).

**Table 1.** Group Mean Data From 17 Participants Showing Slopes and *y*-Intercepts of Linear Regression Models Fitted to Each Participant’s Data in Each of the Three Conditions.

	Slope (*SD*)	Slope > 0	Slope < 1	Intercept (*SD*)	Intercept > 0
Condition 1	0.81 (0.37)	*p* < .001	*p* = .025	2.19 (1.34)	*p* < .001
Condition 2	0.85 (0.32)	*p* < .001	*p* = .013	2.00 (1.20)	*p* < .001
Condition 3	0.76 (0.39)	*p* < .001	*p* = .011	2.19 (1.28)	*p* < .001

*Note.* Group mean slopes and *y*-intercepts were tested for significance using a two-sided Wilcoxon signed-rank test. The effect size *r* values for three conditions are .86, .87, and .87 when testing the intercepts against 0. The *r* values are .88, .88, and .88 when testing the slopes against 0. The *r* values are –.55, –.60, and –.61 when testing the slopes against 1. The results show that there was a bias to overestimate the presented auditory rate in all conditions (intercepts significantly greater than zero) and that the gain in reproduced rates was less than 1.

**Figure 2.**
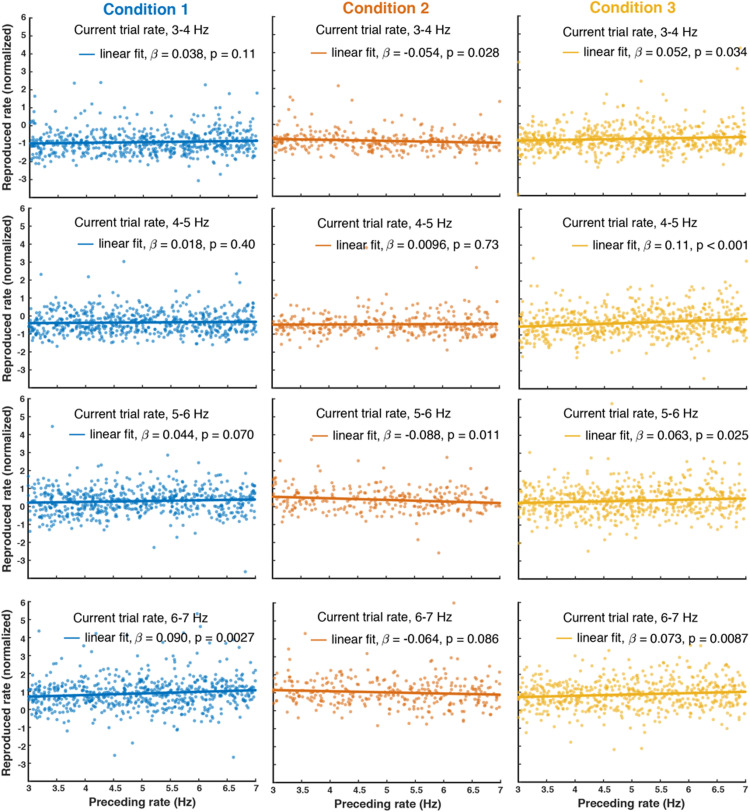
Results from the serial dependence analysis for the three conditions tested showing the influence of the previous trial’s rate on current trial reproduction. Each column shows the aggregated data pooled over all participants in that condition. Trials were divided into four bins based on the frequency presented, 3–4, 4–5, 5–6, or 6–7 Hz (shown in each row), and the data within each bin were modeled with a linear regression to test for the influence of the preceding stimulus rate. The regression lines show how reproduced rates depended on the rate presented on the preceding trial. The legend in each panel shows the slope parameter and the *p*-value of the slope parameter from the linear fitting. In Condition 1, there was a positive dependency on previous rate for the higher frequency bins. In Condition 2, the dependence was negative, and in Condition 3, the dependency was positive. These differences are consistent with predictions and are indicative of the processes underlying serial dependency in rate perception, as discussed in the main text.

To visualize the pattern of serial dependence, we pooled data over observers and conducted a super-subject analysis on the aggregated data, as is commonly used in the field of serial dependence (Fischer & Whitney, 2014; Fritsche et al., 2017). Before pooling observers’ data, each data set was normalized to *z*-scores with a mean 0 and a standard deviation of 1 using MATLAB’S normalize function due to considerable individual differences in both rate bias (regression intercept) and gain (regression slope). The rationale of the serial dependence analysis is as follows. If there is serial dependence, for a given rate on the current trial, its reproduced rates should vary with preceding trial’s rates. Because the rate presented to subjects was randomly chosen in the range of 3–7 Hz, it is not possible to compute serial dependence for each specific rate (e.g., 3.1 Hz, 3.2 Hz, etc.) on the current trial. Thus we grouped rates in a narrow range in which they could be roughly considered similar to test the influence of the preceding trial’s rate. Based on the presented rates, trials were binned into four groups (3–4, 4–5, 5–6, and 6–7 Hz) and we analyzed trials in each group with a linear regression model using MATLAB’S fitglm function to see if the reproduction rate in that group varied as a function of the stimulus rate presented on the preceding trial (i.e., testing whether the regression coefficient β is different from 0). Figure 2 shows the results of this aggregate analysis for the three conditions. In Condition 1, for lower rate bins, the preceding rate did not influence rate perception on the current trial (β = 0.038, standardized β = 0.046, *p* = .11 for 3–4 Hz bin, β = 0.018, standardized β = 0.022, *p* = .40 for 4–5 Hz bin), but there was a positive dependency on preceding rates for the perception of higher rates (β = 0.044, standardized β = 0.054, *p* = .070 for 5–6 Hz bin, β = 0.090, standardized β = 0.11, *p* = .0027 for 6–7 Hz bin).

In Condition 2, where a response was made on every second trial, there was a dominant repulsive dependency on the preceding rates on the perception of current trial rates (β = –0.054, standardized β = –0.069, *p* = .028 for 3–4 Hz bin; β = –0.088, standardized β = –0.11, *p* = .011 for 5–6 Hz bin; β = –0.064, standardized β = –0.082, *p* = .086 for 6–7 Hz bin). In Condition 3, where a longer delay between stimulus and response was used, a significant positive dependency on the preceding rates was found for the perception of all the rate bins on the current trial (β = 0.052, standardized β = 0.062, *p* = .034 for 3–4 Hz bin; β = 0.11, standardized β = 0.13, *p* < .0001 for 4–5 Hz bin; β = 0.063, standardized β = 0.076, *p* < .025 for 5–6 Hz bin; β = 0.073, standardized β = 0.089, *p* = .0087 for 6–7 Hz bin). Our results from the aggregate analysis suggest that auditory rate perception is attracted towards the previously experienced rates, that is, a positive serial dependence. When observers withheld their responses for the preceding stimulus (Condition 2), instead of a positive serial dependency, a repulsive effect occurred, implying that the repulsive dependency happens at the perceptual level and the positive effect happens at a postperceptual level. When observers hold the rates longer in the working memory (Condition 3 vs. Condition 1), the positive serial dependency seems to be stronger, implying that working memory representation of current rates is biased towards the memory trace of previous rates.

**Figure 3.**
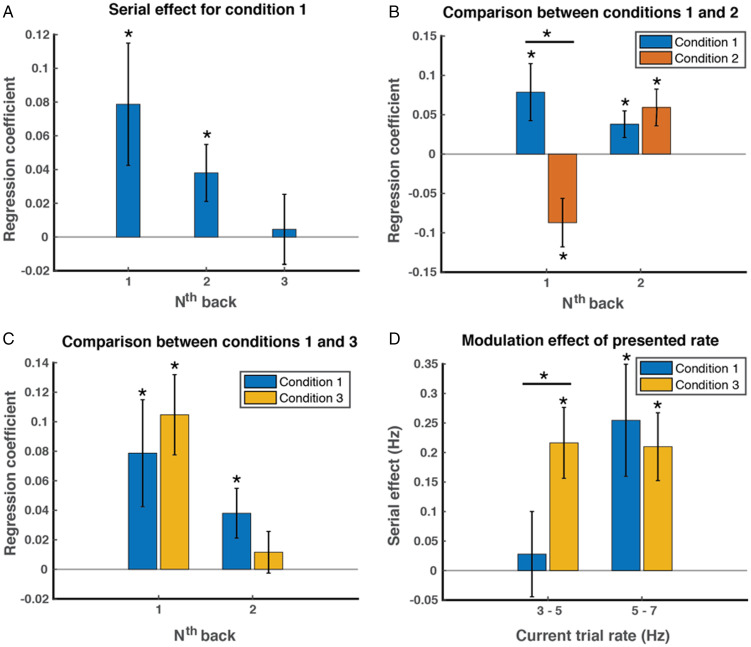
The group average results of serial dependence effect for three conditions. A–C: The serial dependence is computed as the regression coefficient using a linear regression model with reproduced rates as response, and rates presented in preceding trials (1–3 back) as predictor variables. A: The computed magnitude of the serial dependence effect for rates presented one, two, and three trials back from current trial in Condition 1. The positive serial dependency lasts two trials. B: The one-back and two-back serial dependence effect in Conditions 1 and 2. In Condition 2 where observers responded on every alternating trial, a repulsive one-back serial dependence and a positive two-back serial dependence (comparable in magnitude to that in Condition 1) are observed. C: The one-back and two-back serial dependence effect in Conditions 1 and 3. The positive serial dependence is observed for one-back trial rates, but not for two-back trial rates. No strong evidence can support an enhanced serial dependence in Condition 3 than that in Condition 1. D: The influence of current trial rates on the one-back serial dependency in Conditions 1 and 3. For a given range of presented rates (3–5 Hz or 5–7 Hz), the serial dependence is defined as the difference of reproduced rates between trials preceded by 5–7 Hz rates and trials preceded by 3–5 Hz rates. The positive serial dependence is larger in Condition 3 than in Condition 1 when lower rates were presented. The magnitude of serial dependence does not differ for both conditions when higher rates were presented. Color bars are group means with ±1 standard error bars. The * symbols indicate *p* < .05.

The aggregate analysis did not take the random effect caused by variation between subjects into account, so we next conducted a serial dependence analysis for each individual participant and tested for significance at the group level by considering the random effect caused by variation between subjects. We were also interested in how long the serial dependency effect could last since previous studies have shown that the serial dependence effect can still be observed for a few trials back from the present trial (e.g., Fischer & Whitney, 2014; Taubert et al., 2016) as well as the difference between conditions. To this end, for each participant, we fitted a linear regression model with MATLAB’S built-in function fitglm to examine how the reproduced rates varied with the presented rate on the current trial, the presented rate on the one-back trial, the presented rate on the two-back trial, and the presented rate on the three-back trial (four predictors). We obtained regression coefficients of these predictors for each participant. As shown in Figure 3A, for the basic condition (Condition 1), the regression coefficients were significantly larger than 0 (one-sample *t*-test) for the one-back predictor (*M* = 0.079, *SE* = 0.036, *t*(16) = 2.17, Cohen’s *d* = 0.53, *p* = .045) and two-back predictor (*M* = 0.038, *SE* = 0.017, *t*(16) = 2.25, Cohen’s *d* = 0.55, *p* = .039), but not for the three-back predictor (*M* = 0.0046, *SE* = 0.021, *t*(16) = 0.22, Cohen’s *d* = 0.054, *p* = .83), suggesting that the serial dependence effect could last for two trials.

Figure 3B plots the serial dependence effect in Condition 2 with Condition 1 as a reference. One-sample *t*-tests showed that the regression coefficient for the one-back predictor was significantly smaller than 0 (*M* = –0.087, *SE* = 0.031, *t*(16) = –2.83, Cohen’s *d* = –0.69, *p* = .012), but the regression coefficient for the two-back predictor was significantly larger than 0 (*M* = 0.059, *SE* = 0.023, *t*(16) = 2.54, Cohen’s *d* = 0.62, *p* = .022). Note, in Condition 2, the one-back stimuli were not reproduced by participants, but the two-back stimuli were reproduced because of the response alternation design we employed. A further two-way repeated-measures analysis of variance (ANOVA) revealed an interaction between conditions (1 or 2) and *n*-back cases (1 or 2), *F*(1,16) = 8.96, η^2^ = 0.19, *p* =.0086. The simple effects analysis showed that the regression coefficient for the one-back predictor was larger in Condition 1 than that in Condition 2 (paired *t*-test, *t*(16) = 3.61, Cohen’s *d* = 0.87, *p* = .0024), but the two-back regression coefficient did not differ for Condition 1 and Condition 2 (paired *t*-test, *t*(16) = –0.71, Cohen’s *d* = –0.17, *p* = .49). Taken together, in Condition 2, where participants withheld their response every second trial, a repulsive rather than a positive serial dependence on the one-back trial was found, but the positive serial dependence on the two-back trial still remained the same since participants needed to reproduce two-back trials rates in the same way they did in Condition 1. Our results suggest that the repulsive serial effect may reflect a bias at the perceptual level, while the positive serial effect may reflect a bias at a postperceptual level.

In Condition 3, when participants had to hold the rates in working memory longer before their responses, a positive serial effect was found for the one-back case (*M* = 0.10, *SE* = 0.027; *t*(16) = 3.85, Cohen’s *d* = 0.93, *p* = .0014), but not for the two-back case (*M* = 0.012, *SE* = 0.014; *t*(16) = 0.82, Cohen’s *d* = 0.20, *p* = .42), as shown in Figure 3C. It is not surprising that the serial effect only lasts for one trial here because the trial duration is twice as long as the duration in Condition 1. The two-way repeated-measures ANOVA revealed a significant interaction between conditions and *n*-back cases, *F*(1,16) = 4.40, η^2^ = 0.025, *p* = .052, although the simple effects analysis did not show significant differences between conditions 1 and 3 for the one-back case (paired *t*-test, *t*(16) = –1.14, Cohen’s *d* = –0.28, *p* = .27) or the two-back case (*t*(16) = 1.16, Cohen’s *d* = 0.28, *p* = .26).

As shown in Figure 2, our aggregate analysis suggests a serial dependency on preceding rates for the perception of higher rates, but not for lower rates in Condition 1. One possible reason that we did not find a larger serial dependence effect on one-back trial rates in Condition 3 compared to Condition 1 is that the serial effect may already reach the ceiling in Condition 1 for longer rates. Hence, we next examined the influence of current rates on the magnitude of serial dependence on the one-back rates for conditions 1 and 3. We divided each participant’s trials into two groups, one for low presented rates (3–5 Hz) and one for high presented rates (5–7 Hz). For a rate range (3–5 Hz or 5–7 Hz) on the current trial, the serial dependence effect was defined as the reproduced rate difference between trials preceded by the high rates (5–7 Hz) and trials preceded by the low rates (3–5 Hz). Indeed, consistent with the findings in our aggregate analysis, we found significant serial dependency on one-back rates for higher rates (*M* = 0.25, *SD* = 0.095; *t*(16) = 2.68, Cohen’s *d* = 0.65, *p* = .016), but not for lower rates (*M* = 0.028, *SD* = 0.072; *t*(16) = 0.39, Cohen’s *d* = 0.094, *p* = .70) in Condition 1 (Figure 3D).

In Condition 3, one-back serial dependence effect was significantly larger than 0 for both low rate (*M* = 0.22, *SD* = 0.060; *t*(16) = 3.60, Cohen’s *d* = 0.87, *p* = .0024) and high rate (*M* = 0.21, *SD* = 0.058; *t*(16) = 3.65, Cohen’s *d* = 0.89, *p* = .0021), as shown in Figure 3D. A two-way repeated-measures ANOVA showed a significant interaction between conditions (1 vs. 3) and current rate (3–5 vs. 5–7 Hz), *F*(1,16) = 3.91, η^2^ = 0.076, *p* = .065. A further simple effects analysis revealed that for trials where the current rate was low (3–5 Hz), a larger serial dependency was observed in Condition 3 than in Condition 1 (*t*(16) = 2.99, Cohen’s *d* = –0.73, *p* = .0086), but for trials with high rate (5–7 Hz), there was no difference in reproduced rates between two conditions (*t*(16) = 0.45, Cohen’s *d* = 0.11, *p* = .66, paired *t*-test). In sum, we found that in Condition 3 where subjects had to hold the rates in working memory longer before responding, a larger serial dependency on the preceding rate was observed, and this enhancement is more prominent for lower presented rates.

## Discussion

We measured rate perception of isochronous tone sequences in the range of 3–7 Hz using a manual reproduction task. The results in Figure 1 show that, overall, participants were well able to indicate rate in this way as the reproduced rates were linearly related to the presented rates and exhibited a slope that approached unity. An obvious feature of the data is the clear vertical offset in reproduced rates, which were consistently overestimated by about 1 Hz, and slightly more at the low frequencies. The reason for the overestimation is not obvious. Some studies have found that tapping behavior shows an attraction to certain frequencies which are presumably linked to internal oscillators that generate the rhythms of periodic motor activity. For example, one study of unconstrained isochronous tapping found a bimodal distribution with clear peaks at about 2.2 and 3.7 Hz that may represent attractor frequencies (Collyer et al., 1994). However, this account seems unlikely to explain our data as the offset tended to be very consistent rather than showing tuned peaks. Another possibility is that the overestimation could be due to the predictability of isochronous rhythms. Responses to predictable stimuli are faster (Jazayeri & Shadlen, 2015; Miyazaki et al., 2005) and in a reproduction task involving multiple taps, errors may accumulate over the response period to inflate the average rate. Finally, it is not clear whether the overestimation is due to an error in perceiving and remembering the auditory rate or is an error in motor reproduction relative to the remembered rate. Understanding this difference may be important as there is evidence indicating that separate neural systems process timing information for motor and perceptual tasks (Lewis & Miall, 2003; Rao et al., 1997).

A less obvious feature is that the offset is not consistent across the frequency range and the slopes of the regression lines were significantly less than 1. Finding that the slopes were less than unity is consistent with results reported elsewhere of central tendency in time perception. In studies of duration perception, whether the duration was conveyed by visual, auditory, or audio-visual mixed modalities (Acerbi et al., 2012; Cicchini et al., 2012; Jazayeri & Shadlen, 2010; Zhang & Zhou, 2017), reproduction of temporal intervals was shown to exhibit a central tendency towards the mean of the set (i.e., slopes of presented vs. actual durations were significantly less than 1). The data in Figure 1 with their sub-unity slopes are therefore consistent with a central tendency effect in rate perception. The interpretation offered by Jazayeri and Shadlen to explain the central tendency effect is couched in terms of a Bayesian observer which receives noisy stimulus estimates which are combined with a store of information in a prior distribution to obtain the most likely posterior description of the stimulus. The point of Bayesian models like this is that the observed central tendency is not simply an anchoring bias or reference point but is the result of a strategy that combines present and accumulated information in an optimal way (Cicchini et al., 2012; Jazayeri & Shadlen, 2010).

In addition to central tendency, we also measured serial dependence effects in the three conditions. These are evident in the regression fits in Figure 2 and are seen more clearly in the plots shown in Figure 3. The sign of the serial effects is positive in Condition 1, negative in Condition 2 (one-back), and positive again in Condition 3. The only procedural difference between Conditions 1 and 2 was that every trial required a response in Condition 1 whereas in Condition 2 participants only responded on every second trial. There were no differences in stimuli, with an auditory rate stimulus presented on every trial. The shift from positive to negative serial dependence is therefore attributable to the lack of response in Condition 2. This suggests separate components are involved in the standard serial dependence effect (as in Condition 1) where every stimulus is responded to. Withholding responses (Condition 2) reveals a negative (repulsive) perceptual effect, similar to what is seen in classical perceptual aftereffects following adaptation. Repulsive aftereffects like this are well established in the timing domain in other studies exploring the production of perceptual responses without feedback (Becker & Rasmussen, 2007; Maarseveen et al., 2019; Motala et al., 2018; Walker et al., 1981). The fact that making a response to each stimulus, as in Condition 1, produces a positive serial effect implies, first, that there is a positive effect linked to making a perceptual response, and second, that it must be stronger than the stimulus-related repulsive effect because the combined effect of stimulus plus response is positive. Our findings in Condition 1 and Condition 2 suggest that the repulsive effect may originate at the perceptual level while the positive serial dependence occurs at a postperceptual level.

In Condition 3, the time interval between stimulus and response was extended (double the trial length in Condition 1), and it also shows a positive dependency. As in Condition 1, participants made a response on every trial in this condition, again pointing to the response contributing a positive component to the serial dependency. Our finding that the serial effect was stronger in Condition 3 (positive serial effects were found for both low and high rates, see Figure 3D) compared with Condition 1 (positive serial effect only for high rates, see Figure 3D) is consistent with previous findings (Fritsche et al., 2017). It also squares with Bayesian inference which suggests that perception is determined by current sensory evidence and prior knowledge of the context (Jazayeri & Shadlen, 2010; Knill & Pouget, 2004). When the sensory evidence becomes weaker, as in Condition 3 where subjects had to hold the rates in working memory for a longer time, the prior (i.e., the information from the previous trial) would have a greater contribution to the final perception. Again, our findings in Condition 3 strengthen the point that the positive serial dependency originates at a postperceptual level, possibly reflecting a representation bias in working memory.

A central tendency effect describes a characteristic shift of perceptual judgments to be systemically biased towards the mean of the presented distribution. A serial dependence effect describes a tendency for judgments to be influenced by the recent history of stimuli. Nevertheless, it could be argued that central tendency and serial dependence are terms representing the same effect. This was explored in a study using a Bayesian analysis of magnitude estimation (Petzschner et al., 2015). The optimal estimation of magnitude is achieved by combining current sensory evidence and past prior knowledge. In a central tendency effect, a static prior (mean of the stimulus set) is assumed, but a dynamic prior is assumed in the serial dependence effect (prior is adjusted trial-by-trial). The authors consolidate the two effects of perceptual judgments by suggesting that serial dependence effects could also have the potential to create central tendency patterns (Petzschner et al., 2015).

When we react to stimuli, there are different processing stages involved, including perception, decision, and action. A serial dependence may be a universal nature of how our brain operates, hence happening at every stage. In Condition 2 where subjects responded to every alternating trial, we found a repulsive serial dependence on the preceding rate. This is consistent with previous studies showing that perception exhibits repulsive serial dependence (Fritsche et al., 2017; Suárez-Pinilla et al., 2018) to maximize change detection. Consistent with previous findings (Cicchini et al., 2014; Corbett et al., 2011; Manassi et al., 2017), we found positive serial dependence when rates were reproduced every trial and the effect was enhanced when participants had to hold the rates in working memory longer before responding (Condition 3). The absence of positive serial dependence in Condition 2 when responses were withheld suggests that the positive serial dependence happens at a postperceptual stage. Based on the findings in Conditions 2 and 3, serial dependence possibly originates in working memory. Indeed, a recent study shows that a positive serial dependence can be abolished by backward masking, resulting in a repulsive aftereffect, which suggests that high-level modulatory signals are important in forming serial dependency (Fornaciai & Park, 2019). Although we found response was key in forming positive serial dependence, it may be not due to the motor system per se. In fact, there are studies showing that motor responses show repulsive serial dependence, that is, a tendency of alternating motor responses between trials (Pape et al., 2017; Pape & Siegel, 2016; Zhang & Alais, 2020). It is possible that the positive serial dependence is the characteristic of working memory in stabilizing representations of the outside world by integrating information over time (Kiyonaga et al., 2017). Here, it is possible that the requirement of making responses helps transfer sensory information into working memory and strengthens the storage of rates in working memory, hence biasing the subsequent rates reproduction. The overall serial dependence may be the weighted average across these serial effects at each stage (Zhang & Alais, 2020), which could be the reason why previous studies found mixed results of serial dependence being repulsive or positive.

Another factor that could calibrate current decisions is the feedback about previous choices. Indeed, in the decision-making field, a “win-stay-lose-shift” strategy (Nowak & Sigmund, 1993) is extensively observed. Within the framework of Bayesian inference, serial dependence reflects an average of current and previous stimuli weighted according to the uncertainty associated with each experience (Petzschner et al., 2015). Studies on serial dependence reveal that the magnitude of the serial effect can be modulated by the confidence level of previous responses, with high confidence enhancing serial dependence (Braun et al., 2018; Samaha et al., 2019). Feedback can provide extra information about the quality of the decisions on the previous sensory or cognitive stimuli. By providing the correct value of stimulus after each trial, one study found that serial dependence in integer guessing behavior was larger on previous feedback values than on previous responses (Wagner & Baird, 1981). Studies also show that responses can positively depend on each of the several just-past stimuli or responses when feedback is not given, yet when feedback is given, responses are assimilated to the one-back trial but are contrasted with trials further back (Staddon et al., 1980). In a face-attractiveness judgment task, the serial dependence effect was weakened by providing the average attractiveness rating by others for each trial as feedback (Kondo et al., 2012). All these aforementioned studies suggest that feedback reduces the original serial dependence on the previous stimuli or responses. It is possible that feedback could interrupt the continuous trial-by-trial updating of an internal criterion thereby reducing the strength of a serial dependence effect. When subjects integrate information over time, the past history and its quality (evaluated with feedback) are both taken into account. Future work could aim to explore the impact and influence of feedback on serial dependence by manipulating the types of tasks and types of feedback.

These data relate to a current debate in the timing literature concerning whether duration and rate perception are processed by identical or similar mechanisms (Hartcher-O'Brien et al., 2016; Johnston et al., 2006; Keele et al., 1989; Ono & Kitazawa, 2011). A recent study has found evidence for positive serial dependence in auditory duration perception (Roseboom, 2019) and here our findings provide a novel addition in showing that that positive serial dependence also exists for auditory rate perception. This notable similarity between the perception and processing of duration and rate works towards clarifying the relationship between the two. At least on a sensory processing level, observations of positive serial dependence exist for duration and rate independently. To clarify further the extent to which the processing of duration and rate may be interconnected, an interesting further avenue would be to test whether positive serial dependence effects for auditory duration reverse from positive to negative (as found here for Condition 2) when participants withhold motor responses.

In this study, we tested whether rate perception of isochronous tone sequences, measured using a manual rate reproduction task, exhibits a serial dependence effect. We found evidence of positive serial dependence effects in reproduced auditory rates that could be distinguished from an overall central tendency effect also evident in the data. We found evidence that the positive dependence is composed of a small negative component that is stimulus related, and a larger positive component related to response which yields an overall positive effect when a response is required. Thus, while perceived timing can be impressively precise and accurate (Cicchini et al., 2012; Matthews & Meck, 2014), the data reported here show that perceptual reports of successive timing events are not independent and show a tendency to be drawn to the recent stimulus. This effect operates in addition to a central tendency effect (Jazayeri & Shadlen, 2010), also observed here. Although serial and central tendency effects can be regarded as perceptual distortions, both have been argued to be helpful as they help provide perceptual stability (Kiyonaga et al., 2017) and reduce noise by averaging successive noisy stimuli (Cicchini et al., 2018).
